# Monte Carlo dosimetry of the ^60^Co sources of a new GZP3 HDR afterloading system

**DOI:** 10.1186/s13014-023-02207-6

**Published:** 2023-01-20

**Authors:** Junxiang Wu, Shengwei Kang, Pei Wang, Jie Li, Xianliang Wang

**Affiliations:** Sichuan Cancer Hospital and Institute, Sichuan Cancer Center, School of Medicine, University of Electronic Science and Technology of China, Radiation Oncology Key Laboratory of Sichuan Province, Chengdu, 610041 China

**Keywords:** Brachytherapy, ^60^Co source, Monte Carlo, Geant4, EGSnrc, Dosimetry

## Abstract

**Background:**

The purpose of this work was to obtain the dosimetric parameters of the new GZP3 ^60^Co high-dose-rate afterloading system launched by the Nuclear Power Institute of China, which is comprised of two different ^60^Co sources.

**Methods:**

The Monte Carlo software Geant4 and EGSnrc were employed to derive accurate calculations of the dosimetric parameters of the new GZP3 ^60^Co brachytherapy source in the range of 0–10 cm, following the formalism proposed by American Association of Physicists in Medicine reports TG43 and TG43U1. Results of the two Monte Carlo codes were compared to verify the accuracy of the data. The source was located in the center of a 30-cm-radius theoretical sphere water phantom.

**Results:**

For channels 1 and 2 of the new GZP3 ^60^Co afterloading system, the results of the dose-rate constant (Λ) were 1.115 cGy h^−1^ U^−1^ and 1.112 cGy h^−1^ U^−1^, and for channel 3 they were 1.116 cGy h^−1^ U^−1^ and 1.113 cGy h^−1^ U^−1^ according to the Geant4 and EGSnrc, respectively. The radial dose function in the range of 0.25–10.0 cm in a longitudinal direction was calculated, and the fitting formulas for the function were obtained. The polynomial function for the radial dose function and the anisotropy function (1D and 2D) with a $\uptheta$ of 0°–175° and an r of 0.5–10.0 cm were obtained. The curves of the radial function and the anisotropy function fitted well compared with the two Monte Carlo software.

**Conclusion:**

These dosimetric data sets can be used as input data for TPS calculations and quality control for the new GZP3 ^60^Co afterloading system.

## Background

The use of high-dose-rate (HDR) brachytherapy is a highly widespread practice around the world. The International Commission on Radiation Units and Measurement (ICRU) report 89 recommends ^60^Co and ^192^Ir as HDR sources [1]. Because the geometry of the ^192^Ir source is smaller and its energy is lower compared to that of the ^60^Co source, the ^192^Ir source is the primary HDR source in clinical practice. The ^60^Co source results in increased toxic reactions and the issue of radiation protection. Although not as widespread as the ^192^Ir source, the ^60^Co source is also available on remote afterloading systems dedicated to HDR brachytherapy, mainly for the treatment of gynecological cancer [2, 3]. The ^60^Co source has the advantage of a longer half life compared with the ^192^Ir source [4], which provides a longer duration of clinical use and reduced operating costs. Thus, the ^60^Co brachytherapy source is certainly more cost-effective for developing countries [5, 6].

The GZP3 and GZP6 ^60^Co HDR afterloading systems, manufactured by the Nuclear Power Institute of China (NPIC), are widely used at home and abroad. Several studies have been performed on the GZP ^60^Co source [7–9], including the GZP3 and GZP6 models. The dosimetric data for GZP6 60Co source number 3 were obtained by Lei et al. [7] using Monte Carlo (MC)-based dosimetry. Wang et al. [9] calculated the dosimetric data for the GZP3 60Co source using the MC simulation, EGSnrc.

Recently, a new GZP3 ^60^Co HDR afterloading system was manufactured by NPIC, and it has been introduced in brachytherapy procedures. It has three channels in the HDR system; the sources of channels 1 and 2 have the same design while the source of channel 3 has a different design.

According to the recommendations of the American Association of Physicists in Medicine (AAPM) and the European Society for Radiotherapy and Oncology (ESTRO) [10], the dosimetric parameters of all brachytherapy sources must be based on the Radiation Therapy Committee Task Group No. 43 (TG43 and TG43U1) formalism before their clinical use and must be obtained by either experimental or MC methods [11, 12]. Because of the different source designs, the dose distribution for each source model is different; therefore, dosimetric data from different sources cannot be used with each other. MC simulation is widely used in brachytherapy; however, for the new GZP3 ^60^Co HDR sources, there are no dosimetric data available in the literature, based on a comprehensive determination of TG43 and TG43U1.

This study aimed at obtaining the full dosimetric data (i.e., the dose-rate constant, radial dose function and anisotropy function) of the new GZP3 ^60^Co source channels 1, 2 and 3, following the TG43 and TG43U1, using the Monte Carlo codes Geant4 and EGSnrc. The dosimetric data derived in this work were compared with the available data for similar ^60^Co sources. These dosimetric data can be used in treatment planning systems (TPS) as input data and to validate TPS calculations.

## Methods

### Radioactive source structure

**Fig. 1:**
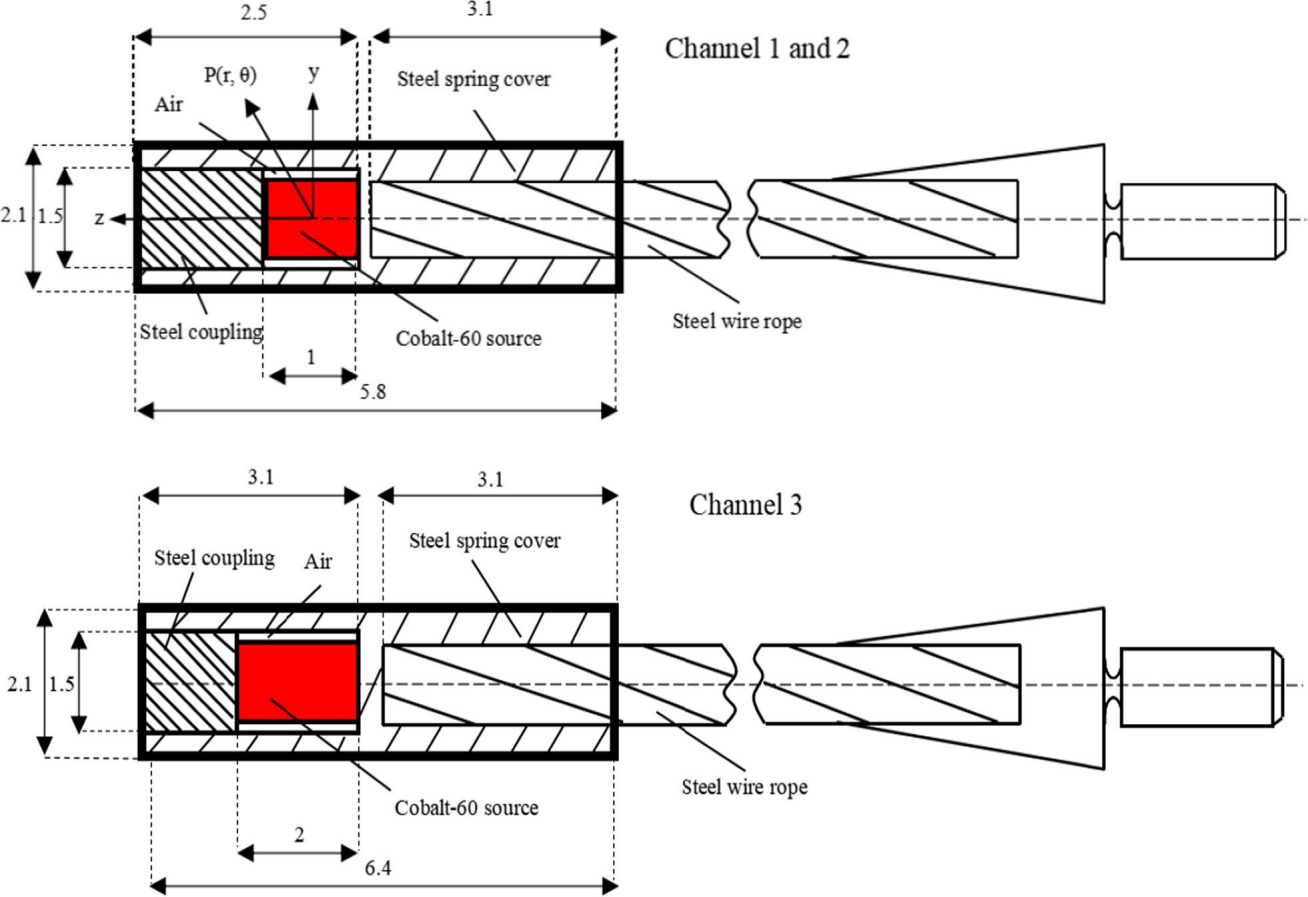
A schematic view of the new GZP3 ^60^Co afterloading system channel 1, 2 and 3. Dimensions are given in mm

The geometric design and materials of the new GZP3 ^60^Co afterloading system were provided by the manufacturer and are shown schematically in Fig. 1. The new GZP3 HDR afterloading system is comprised of two different ^60^Co sources affixed to three channels in the system. It is composed of a central cylindrical active core made of ^60^Co with a density of 8.9 g/cm^3^, 0.5 mm in radius, 1 mm in length for channels 1 and 2, and 2 mm in length for channel 3. The radioactive ^60^Co is distributed uniformly throughout the core. The active core is encapsulated in a 2.1-mm-diameter and 5.8-mm-long cylinder made of stainless steel for channels 1 and 2. The mass density and composition of the materials are shown in Table 1.

**Table 1:** Chemical composition and density of the materials in the Monte Carlo simulations

Material:description	Mass density (g/cm^3^)	Composition (element/weight fraction)
Cobalt:cobalt-60 source	8.85	Co/1
Stainless steel 1 (0Cr18Ni10Ti): steel spring cover, steel coupling	7.98	C/0.001, Si/0.007, Mn/0.01, Cr/0.18, Ni/0.1, Ti/0.004, Fe/0.698
Stainless steel 2 (0Cr18Ni9):steel wire rope	7.93	C/0.001, Si/0.007, Mn/0.01, Cr/0.18, Ni/0.09, Fe/0.712
Air	0.001205	C/0.000124, N/0.755268, O/0.231781, Ar/0.012827
Water:phantom material	1.0	H/0.111901, O/0.888099

### Dose-calculation formalism

The 1D and 2D dose calculation formalism proposed by the AAPM TG43 report was followed [11]. According to this formalism, the 1D equation used to calculated dose rate, as shown in Eqs. (1). The 2D dose rate, ${\dot{\text{D}}}$(r, θ), at point (r, θ) in the medium, where r is the distance in cm form the active source center and θ is the polar angle relative to the longitudinal axis of the source, as shown in Eqs. (2):

1 $${\dot{\text{D}}}\left( {\text{r}} \right){\text{ = S}}_{{\text{k}}} { }\Lambda \frac{{{\text{G(r,}}\uptheta_{{0}}{)}}}{{{\text{G(r}}_{{0}} {,}\uptheta _{{0}} {)}}}{\text{g(r) }}\phi_{{{\text{an}}}} {\text{(r)}}$$

2 $${\dot{\text{D}}}\left( {{\text{r,}}\uptheta } \right){\text{ = S}}_{{\text{k}}} { }\Lambda \frac{{{\text{G(r,}}\uptheta {)}}}{{{\text{G(r}}_{{0}} {,}\uptheta _{{0}} {)}}}{\text{g(r) F(r,}}\uptheta {)}$$

where S_k_ is the source-air-kerma strength in units of U (1U = 1 μGy m^2^ h^−1^ = 1 cGy cm^2^ h^−1^). The reference distance r_0_ and angle θ_0_ are 1 cm and π/2, respectively.

Λ is the dose-rate constant defined as the ratio of dose rate to water at the reference point (r_0_, θ_0_) and air-kerma strength,

3 $$\Lambda = {\dot{\text{D}}}\left( {{\text{r}}_{0} ,\uptheta _{0} } \right)/{\text{S}}_{{\text{k}}}$$

G(r,θ) is the geometry factor, defined as

4 $${\text{G(r, }}\uptheta {) = }\left( {\frac{\upbeta }{{{\text{L rsin}}\uptheta }}} \right)$$

where L is the active length of the source, and β is the angle subtended by the active source with respect to the point (r,θ).

The value g(r) is the radial dose function, defined as

5 $${\text{g}}({\text{r}}) = \frac{{{\dot{\text{D}}}({\text{r}},\uptheta _{0} ){\text{G}}({\text{r}}_{0} ,\uptheta _{0} )}}{{{\dot{\text{D}}}({\text{r}}_{0} ,\uptheta _{0} ) {\text{G}}({\text{r}},\uptheta _{0} )}}$$

The value $\phi_{{{\text{an}}}} {\text{(r)}}$ is the 1D anisotropy function, defined as

6 $$\phi_{{{\text{an}}}} \left( {\text{r}} \right) = \frac{{\mathop \smallint \nolimits_{0}^{\pi } {\dot{\text{D}}}({\text{r}},\uptheta )\sin (\uptheta ){\text{d}}\uptheta }}{{2{\dot{\text{D}}}({\text{r}},\uptheta _{0} )}}$$

and F(r,θ) is the 2D dose-anisotropy function, defined as

7 $${\text{F}}({\text{r}},\uptheta ) = \frac{{{\dot{\text{D}}}({\text{r}},\uptheta ){\text{G}}({\text{r}},\uptheta _{0} )}}{{{\dot{\text{D}}}({\text{r}},\uptheta _{0} ){\text{G}}({\text{r}},\uptheta )}}$$

### Monte Carlo calculations

In this study, we employed the Monte Carlo codes Geant4 (version 10.4) [13] and EGSnrc [14] to derive all dosimetric parameters of the new GZP3 ^60^Co sources, following the formalism described by TG43 and TG43U1. These MC codes have been successfully and widely used in dosimetric studies of brachytherapy [15–18]. Different MC codes used different physics models and different cross-sections of data in the transport of electrons. The low-energy physics model of Geant4 was used. This physics model uses the EPDL97 cross sections [19] for photons and EEDL [20] for electrons. For the EGSnrc, the photon cross sections from the XCOM database [21] were used. The cutoff energy was set to 10 keV for both photons and electrons. The ^60^Co spectrum was obtained from the NuDat database, taking only the two gamma photons with 1.17 and 1.33 MeV of energy in the simulation [22]. The contribution of the β spectrum to the dose was not considered in the simulation due to the absorption with stainless steel cover around the metallic ^60^Co, with the total dose of electrons less than 1% at distances greater than 1.0 mm from the source [23, 24].

In order to obtain the dose distribution in water, the ^60^Co source was located at the center of a spherical liquid water phantom with a 30-cm radius in the simulation, which acted as an unbounded phantom [25]. Three different grid sizes were used to score the absorbed dose at distances from the center of the source r ranging from 0.25 to 10.0 cm and polar angles ranging from 5° to 180°. The first was composed of cylindrical rings 0.025 cm thick and 0.005 cm high, for 0 cm < r ≤ 1.0 cm; the second was composed of cylindrical rings 0.05 cm thick and 0.05 cm high, for 1 cm < r < 3.0 cm; and the third one was composed of cylindrical rings 0.1 cm thick and 0.1 cm high, for 3.0 cm ≤ r ≤ 10.0 cm. In the coordinate system, θ, y, and z are representative of the polar angle and radial and axial coordinates, respectively. The coordinate axes used are shown in Fig. 1. The dose absorbed in the water was calculated in Geant4 using the function of GetTotalEnergyDeposit, whereas in EGSnrc the tally afforded within the DOSRZnrc package was used. The absorbed dose were calculated to obtained radial dose function and anisotropy function in spherical (r, θ) and cylindrical (y, z) coordinates. In spherical coordinates, r is defined as the distance from the center of the source. In cylindrical coordinates, y and z are representative of the radial and axial coordinates, respectively. When z = 0, we set up the cylindrical rings at y ranging from 0.25 cm to 10 cm to obtain the absorbed dose D(y, z), and then used equation g(r) to calculated the radial dose function. Simultaneously, we set up cells at the same distances as those used in the cylindrical coordinate system (0.25 cm ≤ r ≤ 10.0 c), and θ ranged from 5˚ to 180˚ while obtaining the absorbed dose D(r, θ), and then we calculated the anisotropy function by equation F(r, θ).

Due to the high energy of the ^60^Co, electronic equilibrium was reached at a distance of about 1.0 cm. The differences between dose and kerma were 1.5% at 0.5 cm from the source, less than 0.5% at 0.7 cm and negligible at distances greater than 1.0 cm [23]. Thus, the dose cannot be approximated by kerma at very small distances (< 0.5 cm), unlike ^192^Ir and ^137^Cs sources. In order to accelerate calculations and reduce statistical uncertainty and the computation time, kerma was obtained for r > 0.7 cm. To estimate the air-kerma strength, the ^60^Co source was located in the center of a 3 × 3 × 3-m-cubed air phantom. As TG43U1 recommended [12], the air used in this simulation had 0% humidity. The cylindrical ring was used to score for kerma of 0.1 cm in height and 0.1 cm in diameter.

The number of photon histories was 5 × 10^9^ to obtain dose values and 2 × 10^9^ photon histories to obtain kerma values.

## Results and discussion

### Dose-rate constant

The air-kerma strength, S_k_, was calculated by using Geant4 and EGSnrc. Its values were 2.985 × 10^–7^ and 2.981 × 10^–7^ cGy cm^2^ h^−1^ Bq^−1^ for channels 1 and 2, and 2.975 × 10^–7^ and 2.969 × 10^–7^ cGy cm^2^ h^−1^ Bq^−1^ for channel 3. The dose-rate constant for the ^60^Co source were found to be equal to 1.115 ± 0.004 and 1.112 ± 0.004 cGy h^−1^ U^−1^ for channels 1 and 2 and 1.116 ± 0.004 and 1.113 ± 0.004 cGy h^−1^ U^−1^ for channel 3. These results are presented in Table 2, along with the corresponding values calculated for the similar ^60^Co HDR sources. The difference between the dose-rate constants of channels 1, 2 and 3 with two Monte Carlo codes were 0.27% and 0.27%, respectively. Furthermore, Table 2 shows that all the dose-rate-constant values with different sources matched closely (within 2.87%) and the values of the new GZP3 ^60^Co sources were slightly higher than the other types (GZP6, BEBIG and Flexisource) of ^60^Co sources.

**Table 2:** Comparison of dose rate constant values for similar ^60^Co sources

Source design	Λ (cGy h^−1^ U^−1^)
New GZP3 ^60^Co Channel 1 and 2 (This work by Geant4)	1.115 ± 0.004
New GZP3 ^60^Co Channel 1 and 2 (This work by EGSnrc)	1.112 ± 0.004
New GZP3 ^60^Co Channel 3 (This work by Geant4)	1.116 ± 0.004
New GZP3 ^60^Co Channel 3 (This work by EGSnrc)	1.113 ± 0.004
GZP6 ^60^Co (MCNP) [8]	1.104 ± 0.003
BEBIG ^60^Co (Geant4) [24]	1.084 ± 0.005
Flexisource ^60^Co (Geant4) [26]	1.085 ± 0.003

### Radial dose functions and anisotropy functions

**Fig. 2:**
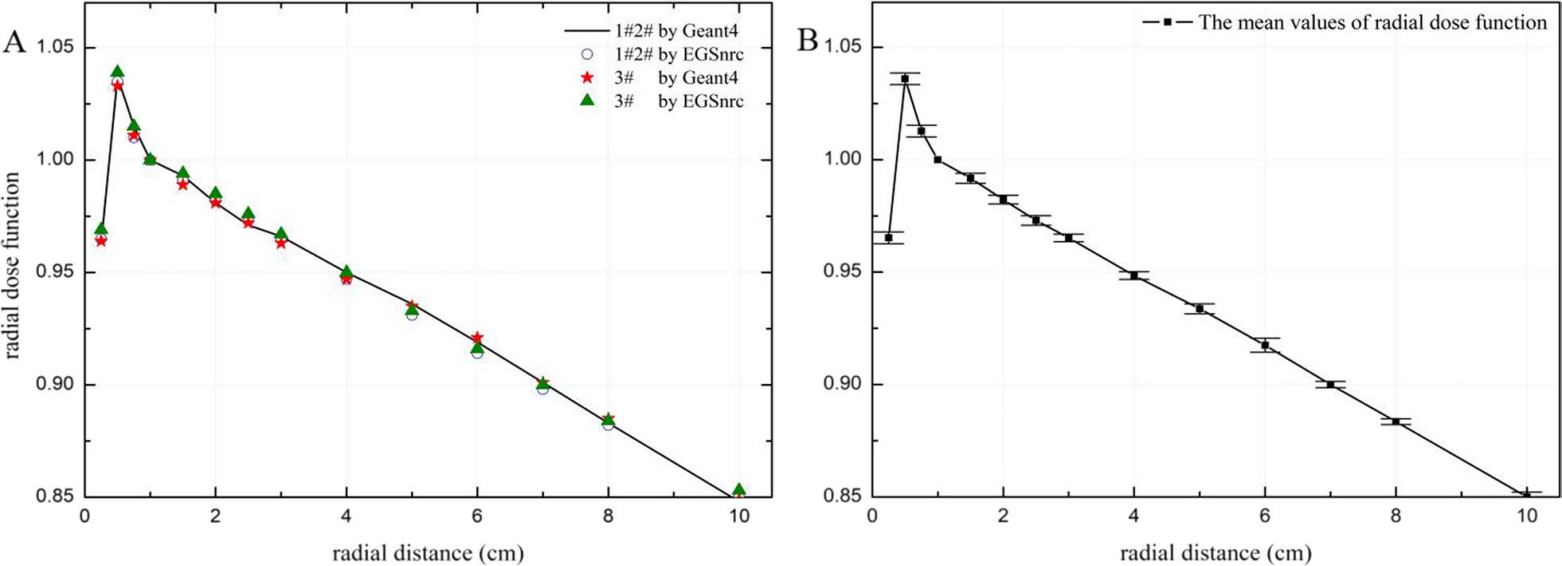
**A** Radial dose function of the new ^60^Co afterloading system with Geant4 and EGSnrc. **B** The error bars of Geant4 and EGSnrc for radial dose function

**Fig. 3:**
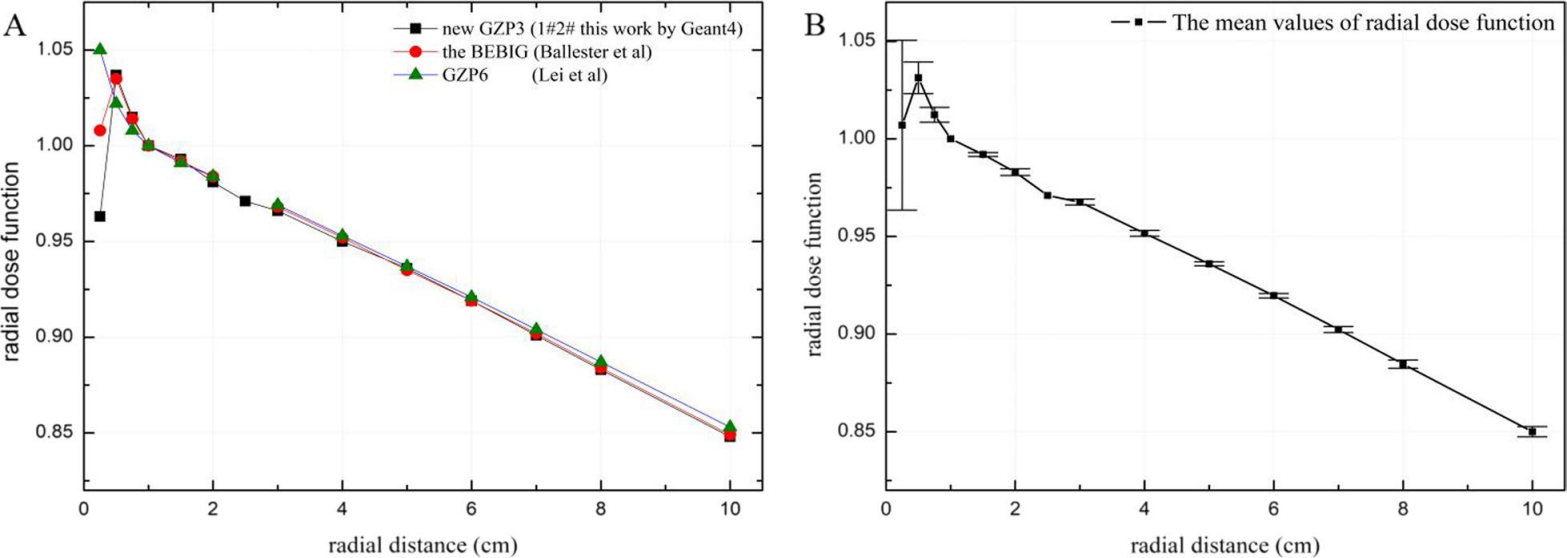
**A** Radial dose function of ^60^Co source models. **B** The error bars among ^60^Co source models for radial dose function

The values of the radial dose function g(r) of the new GZP3 ^60^Co source for radial distances from 0.25 to 10.0 cm by two MC codes are presented in Table 3. Table 3 and Fig. 2 show that the g(r) values between the two MC codes match well; the differences were less than 0.55% for channels 1 and 2 and less than 0.58% for channel 3. The differences in Fig. 2 were calculated as follow: (g_EGSnrc_-g_Geant4_)/g_Geant4_, where g_EGSnrc_ and g_Geant4_ represent the values of the radial dose function for the EGSnrc and Geant4, respectively. The radial dose function obtained for the new GZP3 ^60^Co source is compared with similar sources from the relevant literature in Fig. 3. The differences in Fig. 3 were calculated as follow: (g_GZP3_-g_others_)/g_GZP3_, where g_GZP3_ and g_others_ represent the values of the radial dose function for this work by Geant4 and others work, respectively. This comparison shows that the curves of the g(r) were very similar at r > 1 cm and small differences were present at r < 1 cm. The results of our calculations of channels 1 and 2 were compared with those of Ballester et al. [24] for the BEBIG ^60^Co source by Geant4: for 0.25 ≤ r ≤ 1.0 cm, the differences were less than 4.64% and for 1 < r ≤ 10.0 cm, they were less than 0.32%. These differences were due to varying degrees of photon scattering and absorption in the sources, while the differences in the dimensions and encapsulation designs of the sources were negligible [27, 28].

**Table 3:** Radial dose function calculated for the new GZP3 ^60^Co afterloading system by Geant4 and EGSnrc

r (cm)	g(r)			
	Channel 1 and 2		Channel 3	
	Geant4	EGSnrc	Geant4	EGSnrc
0.25	0.963	0.965	0.964	0.969
0.5	1.037	1.035	1.033	1.039
0.75	1.015	1.010	1.011	1.015
1	1.000	1.000	1.000	1.000
1.5	0.991	0.991	0.989	0.994
2	0.981	0.982	0.981	0.985
2.5	0.971	0.973	0.972	0.976
3	0.966	0.965	0.963	0.967
4	0.950	0.947	0.947	0.950
5	0.936	0.931	0.935	0.933
6	0.917	0.914	0.921	0.916
7	0.898	0.898	0.901	0.900
8	0.883	0.882	0.885	0.884
10	0.852	0.850	0.849	0.853

The radial dose functions obtained by Geant4 were fitted to a fifth-order polynomial function over the range of 0.25 ≤ r ≤ 10.0 cm according to:

$${\text{g}}\left( {\text{r}} \right) = \left( {{\text{a}}0{\text{r}}^{ - 2} + {\text{a}}1{\text{r}}^{ - 1} + {\text{a}}2 + {\text{a}}3{\text{r}} + {\text{a}}4{\text{r}}^{2} } \right){\text{e}}^{{ - a5{\text{r}}}}$$

The values of the fitted parameters were as follows: (a) for channel 1 and 2, R^2^ = 0.995, a0 = − 0.023, a1 = 0.112, a2 = 0.885, a3 = − 0.112, a4 = 0.004 and a5 = 0.149; and (b) for channel 3, R^2^ = 0.995, a0 = − 0.022, a1 = 0.102, a2 = 0.895, a3 = − 0.11, a4 = 0.004 and a5 = 0.142. The overall accuracy of the fits was excellent; the maximum deviations were less than 0.46% and 0.41% for channels 1, 2 and 3, respectively.

**Fig. 4:**
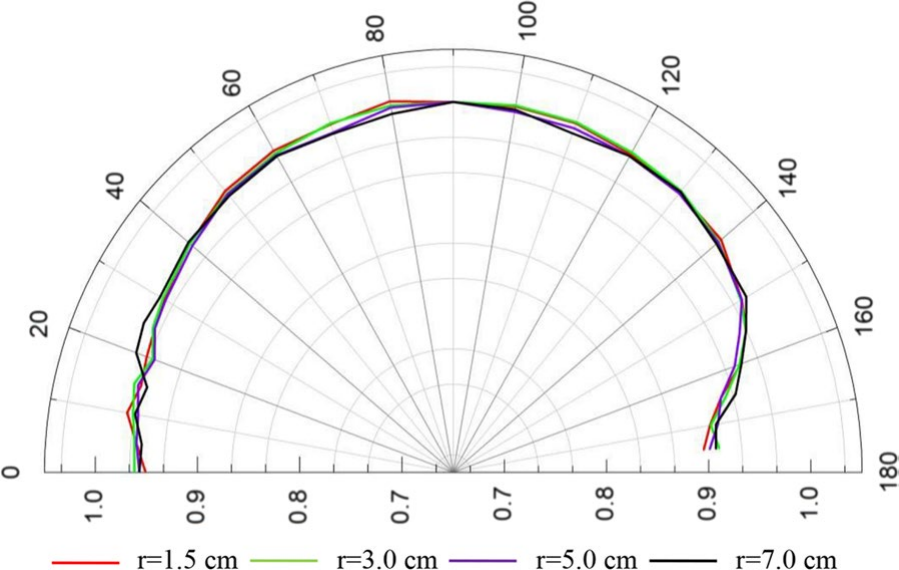
Anisotropy function of the new GZP3 ^60^Co afterloading system with channel 3 at selected distances

In Tables 4 and 5, the full data for the 1D and 2D anisotropy functions by Geant4 and F(r, θ) are presented at radial distances r = 0–10.0 cm and at polar angles θ = 0°–175°. The F(r, θ) values for the selected radial distances of 1.5 cm, 3.0 cm, 5.0 cm and 7.0 cm are presented in Fig. 4. In this figure, it can be seen that, at the polar angles close to the long axis, the values of the F(r, θ) presented a non-smooth shape with F(r, θ) values significantly lower than the other angles (30° ≤ θ ≤ 150°), which was due to the oblique filtration within the source design.

**Table 4:** 1D and 2D anisotropy function calculated for the new GZP3 ^60^Co afterloading system of Channel 1 and 2 by Geant4

θ(°)	r (cm)													
	0.25	0.5	0.75	1.0	1.5	2.0	2.5	3.0	4.0	5.0	6.0	7.0	8.0	10.0
0	0.717	0.945	0.951	0.950	0.954	0.928	0.932	0.969	0.952	0.936	0.940	0.968	0.946	0.935
5	0.711	0.941	0.954	0.960	0.961	0.954	0.975	0.965	0.978	0.964	0.949	0.970	0.946	0.958
10	0.705	0.948	0.956	0.967	0.965	0.970	0.973	0.971	0.964	0.969	0.968	0.978	0.955	0.960
15	0.787	0.942	0.953	0.963	0.963	0.964	0.967	0.968	0.968	0.966	0.970	0.973	0.962	0.968
20	0.760	0.942	0.967	0.972	0.962	0.966	0.969	0.963	0.961	0.959	0.967	0.974	0.969	0.967
25	0.733	0.947	0.960	0.972	0.963	0.969	0.961	0.970	0.970	0.961	0.973	0.977	0.970	0.965
30	0.774	0.948	0.970	0.977	0.964	0.974	0.973	0.969	0.975	0.970	0.968	0.975	0.976	0.981
40	0.803	0.972	0.983	0.990	0.980	0.979	0.984	0.979	0.986	0.985	0.977	0.982	0.991	0.984
50	0.884	0.984	0.987	0.987	0.988	0.994	0.987	0.991	0.988	0.991	0.991	0.986	0.993	0.990
60	0.941	0.994	0.994	0.994	0.992	0.996	0.992	0.996	0.993	0.993	0.995	0.996	1.000	0.994
70	0.972	0.998	0.991	1.002	0.992	0.993	0.999	0.998	1.002	0.995	1.000	0.994	1.002	1.000
80	0.991	1.002	0.995	1.000	0.995	0.998	1.002	1.001	1.000	0.997	0.998	1.000	1.000	1.000
90	1.000	1.000	1.000	1.000	1.000	1.000	1.000	1.000	1.000	1.000	1.000	1.000	1.000	1.000
100	0.995	0.997	0.996	1.004	0.999	0.998	1.001	1.000	1.003	0.999	0.999	1.001	1.003	0.998
110	0.978	0.998	1.000	0.995	0.996	0.997	0.999	1.001	0.998	0.993	0.999	0.994	1.002	1.002
120	0.949	0.994	0.994	0.998	0.993	0.994	0.994	0.995	0.998	0.998	0.995	0.998	0.994	0.995
130	0.921	0.983	0.991	0.993	0.987	0.995	0.990	0.994	0.991	0.987	0.990	0.994	0.995	0.994
140	0.834	0.977	0.979	0.992	0.982	0.983	0.984	0.983	0.985	0.981	0.981	0.985	0.985	0.983
150	0.825	0.945	0.968	0.969	0.967	0.975	0.975	0.972	0.973	0.968	0.973	0.975	0.973	0.972
155	–	0.912	0.950	0.959	0.954	0.962	0.963	0.961	0.961	0.961	0.960	0.959	0.962	0.969
160	–	0.859	0.947	0.946	0.937	0.945	0.949	0.948	0.941	0.949	0.945	0.947	0.943	0.939
165	–	0.802	0.921	0.927	0.921	0.922	0.924	0.927	0.932	0.931	0.928	0.935	0.932	0.933
170	–	–	0.915	0.923	0.907	0.915	0.918	0.920	0.925	0.925	0.919	0.917	0.927	0.924
175	–	–	–	–	–	0.929	0.922	0.920	0.920	0.923	0.927	0.928	0.907	0.931
ϕ_an_(r)	–	–	–	–	–	0.993	0.994	0.994	0.994	0.992	0.993	0.994	0.996	0.994

**Table 5:** 1D and 2D anisotropy function calculated for the new GZP3 ^60^Co afterloading system of Channel 3 by Geant4

θ(°)	r (cm)													
	0.25	0.5	0.75	1.0	1.5	2.0	2.5	3.0	4.0	5.0	6.0	7.0	8.0	10.0
0	0.698	0.943	0.954	0.965	0.951	0.930	0.930	0.962	0.937	0.957	0.949	0.957	0.946	0.932
5	0.697	0.944	0.968	0.968	0.961	0.960	0.965	0.963	0.970	0.961	0.958	0.956	0.951	0.955
10	0.687	0.947	0.966	0.975	0.974	0.968	0.962	0.968	0.966	0.963	0.956	0.966	0.952	0.972
15	0.771	0.952	0.973	0.973	0.966	0.962	0.964	0.973	0.971	0.969	0.961	0.960	0.961	0.973
20	0.741	0.945	0.969	0.970	0.969	0.971	0.974	0.963	0.975	0.961	0.966	0.980	0.965	0.972
25	0.799	0.951	0.975	0.980	0.972	0.975	0.971	0.974	0.970	0.972	0.970	0.984	0.972	0.972
30	0.770	0.959	0.977	0.981	0.977	0.981	0.979	0.978	0.980	0.975	0.972	0.981	0.976	0.969
40	0.806	0.973	0.984	0.984	0.986	0.984	0.986	0.986	0.991	0.983	0.978	0.988	0.985	0.988
50	0.892	0.992	0.988	0.986	0.997	0.995	0.993	0.994	0.995	0.993	0.984	0.991	0.989	0.992
60	0.945	0.996	0.991	0.996	1.001	1.005	0.993	0.998	1.000	0.996	0.989	0.995	0.993	0.995
70	0.978	1.000	0.999	0.998	1.000	1.000	0.998	1.001	1.000	0.992	0.990	0.991	0.993	0.993
80	0.995	1.004	1.000	0.999	1.006	1.331	1.002	1.002	1.001	1.000	0.992	0.994	1.000	1.000
90	1.000	1.000	1.000	1.000	1.000	1.000	1.000	1.000	1.000	1.000	1.000	1.000	1.000	1.000
100	0.994	1.001	1.000	1.003	1.001	1.332	1.004	1.002	1.001	0.996	0.994	0.998	0.995	0.997
110	0.980	0.998	0.995	0.998	1.001	1.004	1.000	1.002	1.002	0.996	0.987	0.991	0.998	0.999
120	0.951	0.999	0.991	0.994	0.997	1.000	0.999	0.999	0.996	0.995	0.990	0.995	0.995	0.993
130	0.931	0.995	0.991	0.989	0.995	0.996	0.993	0.997	0.996	0.994	0.983	0.996	0.993	0.988
140	0.839	0.985	0.988	0.986	0.992	0.996	0.992	0.989	0.987	0.988	0.981	0.986	0.982	0.988
150	0.835	0.958	0.978	0.973	0.976	0.980	0.979	0.975	0.977	0.976	0.971	0.981	0.973	0.977
155	–	0.923	0.958	0.964	0.966	0.966	0.969	0.966	0.968	0.959	0.959	0.966	0.966	0.966
160	–	0.864	0.952	0.942	0.949	0.951	0.947	0.948	0.950	0.943	0.949	0.950	0.947	0.953
165	–	0.774	0.919	0.920	0.921	0.934	0.929	0.926	0.929	0.921	0.923	0.936	0.934	0.937
170	–	–	0.899	0.907	0.905	0.910	0.911	0.906	0.912	0.913	0.903	0.911	0.916	0.937
175	–	–	–	–	0.896	0.902	0.904	0.911	0.904	0.902	0.895	0.908	0.906	0.913
ϕ_an_(r)	–	–	–	–	0.999	0.999	0.997	0.997	0.997	0.993	0.988	0.994	0.993	0.994

### Dose-rate distribution and uncertainties

The along–away 2D-dose-rate results (cGy h^−1^ U^−1^) for the channel 1, and 2 and 3 ^60^Co source for quality assurance (QA) purposes are presented in Tables 6 and 7, respectively. The QA values were tabulated over the ranges of − 10 cm ≤ z ≤ 10 cm and 0 ≤ y ≤ 10 cm.

**Table 6:** Dose rate results (cGy h ^−1^ U ^−1^), for the new GZP3 ^60^Co afterloading system of Channel 1 and 2 by Geant4

z(cm)	y(cm)													
	0	0.25	0.5	0.75	1.0	1.5	2.0	3.0	4.0	5.0	6.0	7.0	8.0	10.0
− 10	0.030	0.030	0.030	0.030	0.030	0.030	0.030	0.028	0.027	0.024	0.022	0.020	0.018	0.014
− 8	0.051	0.050	0.050	0.050	0.048	0.048	0.047	0.044	0.039	0.035	0.031	0.027	0.024	0.018
− 7	0.066	0.066	0.066	0.066	0.064	0.064	0.061	0.056	0.050	0.043	0.038	0.032	0.028	0.020
− 6	0.096	0.092	0.092	0.091	0.088	0.088	0.083	0.073	0.063	0.054	0.045	0.038	0.032	0.023
− 5	0.135	0.135	0.135	0.134	0.128	0.124	0.116	0.099	0.082	0.067	0.054	0.044	0.036	0.025
− 4	0.218	0.213	0.214	0.208	0.198	0.189	0.172	0.138	0.107	0.083	0.064	0.051	0.041	0.027
− 3	0.377	0.386	0.376	0.365	0.348	0.312	0.269	0.196	0.139	0.102	0.076	0.057	0.045	0.029
− 2	0.854	0.883	0.840	0.771	0.716	0.571	0.449	0.274	0.177	0.120	0.085	0.064	0.048	0.031
− 1.5	1.600	1.545	1.429	1.275	1.113	0.809	0.578	0.319	0.194	0.127	0.090	0.066	0.050	0.031
− 1	3.451	3.405	2.899	2.336	1.845	1.124	0.731	0.362	0.209	0.135	0.093	0.067	0.051	0.031
− 0.75	6.703	5.825	4.517	3.321	2.385	1.309	0.807	0.380	0.216	0.137	0.095	0.068	0.052	0.032
− 0.5	14.964	11.884	7.533	4.627	2.987	1.481	0.862	0.389	0.220	0.139	0.095	0.069	0.052	0.032
0	–	57.034	15.555	6.778	3.749	1.656	0.919	0.402	0.223	0.140	0.096	0.069	0.052	0.032
0.5	–	11.653	7.534	4.643	3.001	1.483	0.868	0.393	0.220	0.139	0.095	0.069	0.052	0.032
0.75	–	5.672	4.537	3.316	2.386	1.314	0.805	0.379	0.215	0.137	0.094	0.068	0.051	0.032
1	–	3.313	2.891	2.360	1.838	1.130	0.733	0.362	0.210	0.135	0.093	0.068	0.051	0.032
1.5	–	1.488	1.395	1.270	1.111	0.807	0.578	0.321	0.194	0.128	0.090	0.066	0.050	0.031
2	–	0.831	0.798	0.764	0.704	0.577	0.450	0.273	0.176	0.120	0.085	0.064	0.049	0.031
3	–	0.368	0.364	0.346	0.342	0.311	0.270	0.193	0.140	0.101	0.075	0.058	0.045	0.029
4	–	0.206	0.202	0.198	0.145	0.184	0.171	0.138	0.107	0.083	0.065	0.051	0.041	0.027
5	–	0.127	0.126	0.126	0.093	0.120	0.114	0.198	0.083	0.067	0.054	0.044	0.036	0.025
6	–	0.087	0.087	0.087	0.064	0.083	0.080	0.073	0.063	0.054	0.045	0.038	0.032	0.023
7	–	0.065	0.064	0.062	0.047	0.061	0.059	0.055	0.050	0.044	0.038	0.032	0.027	0.020
8	–	0.049	0.047	0.048	0.035	0.046	0.045	0.043	0.039	0.035	0.031	0.027	0.024	0.018
10	–	0.029	0.029	0.030	0.022	0.029	0.028	0.027	0.026	0.024	0.022	0.020	0.018	0.014

**Table 7:** Dose rate results (cGy h ^−1^ U ^−1^), for the new GZP3 ^60^Co afterloading system of Channel 3 by Geant4

z(cm)	y(cm)													
	0	0.25	0.5	0.75	1.0	1.5	2.0	3.0	4.0	5.0	6.0	7.0	8.0	10.0
− 10	0.032	0.032	0.031	0.030	0.030	0.030	0.030	0.028	0.026	0.024	0.022	0.020	0.018	0.014
− 8	0.048	0.050	0.049	0.049	0.048	0.048	0.047	0.044	0.040	0.036	0.032	0.027	0.024	0.018
− 7	0.065	0.068	0.066	0.066	0.064	0.064	0.062	0.056	0.050	0.044	0.038	0.032	0.028	0.020
− 6	0.089	0.093	0.093	0.091	0.087	0.087	0.083	0.074	0.063	0.054	0.045	0.038	0.032	0.023
− 5	0.133	0.134	0.133	0.133	0.127	0.125	0.116	0.100	0.082	0.067	0.054	0.044	0.036	0.025
− 4	0.216	0.215	0.212	0.208	0.197	0.189	0.174	0.138	0.107	0.083	0.064	0.051	0.041	0.027
− 3	0.347	0.387	0.380	0.365	0.351	0.313	0.272	0.196	0.139	0.101	0.076	0.058	0.045	0.029
− 2	0.850	0.876	0.836	0.779	0.716	0.575	0.449	0.274	0.177	0.120	0.086	0.063	0.048	0.031
− 1.5	1.568	1.561	1.439	1.288	1.114	0.812	0.580	0.319	0.194	0.128	0.090	0.066	0.050	0.031
− 1	3.695	3.400	2.959	2.385	1.870	1.131	0.731	0.361	0.209	0.135	0.093	0.067	0.051	0.032
− 0.75	6.433	5.947	4.595	3.305	2.382	1.310	0.803	0.376	0.214	0.137	0.094	0.068	0.051	0.032
− 0.5	15.054	12.252	7.608	4.589	3.006	1.484	0.865	0.391	0.220	0.139	0.095	0.069	0.052	0.032
0	–	55.069	15.334	6.716	3.775	1.649	0.918	0.403	0.223	0.141	0.096	0.069	0.052	0.032
0.5	–	12.025	7.606	4.602	2.995	1.483	0.864	0.391	0.220	0.139	0.095	0.069	0.052	0.032
0.75	–	5.862	4.605	3.334	2.395	1.313	0.802	0.378	0.215	0.137	0.094	0.068	0.051	0.032
1	–	3.254	2.920	2.378	1.871	1.129	0.736	0.359	0.209	0.135	0.093	0.068	0.051	0.032
1.5	–	1.472	1.396	1.274	1.123	0.809	0.582	0.319	0.194	0.127	0.090	0.066	0.050	0.031
2	–	0.826	0.798	0.763	0.712	0.577	0.450	0.275	0.176	0.120	0.086	0.063	0.048	0.030
3	–	0.367	0.354	0.349	0.342	0.311	0.271	0.195	0.140	0.102	0.075	0.058	0.045	0.029
4	–	0.199	0.198	0.196	0.144	0.184	0.173	0.139	0.107	0.083	0.065	0.051	0.040	0.027
5	–	0.128	0.125	0.125	0.090	0.120	0.115	0.198	0.082	0.067	0.054	0.044	0.036	0.025
6	–	0.086	0.086	0.085	0.063	0.083	0.081	0.074	0.064	0.054	0.045	0.038	0.032	0.022
7	–	0.062	0.063	0.063	0.046	0.061	0.059	0.055	0.050	0.044	0.038	0.032	0.028	0.020
8	–	0.047	0.046	0.046	0.035	0.046	0.045	0.043	0.039	0.035	0.032	0.028	0.024	0.018
10	–	0.030	0.029	0.029	0.021	0.029	0.028	0.027	0.026	0.024	0.022	0.020	0.018	0.014

The uncertainties in the final dose-rate distributions for the ^60^Co sources, including statistical uncertainty (type A) and systematic uncertainty (type B), were calculated as recommended by TG43U1.^9^ The statistical uncertainty of the ^60^Co source depended on the axial position z and the longitudinal position y. In this study, the statistical uncertainty was less than 0.72% and 0.73% for the points located on or very near to the longitudinal axis and less than 1.21% and 1.19% for the other points, for channels 1, 2, and 3 by Geant4, respectively.

For the new ^60^Co source, the main source of a systematic uncertainty based on the Geant4 simulation was the source geometry. The tolerances of this new source to the active core diameter were ± 0.05 mm, as provided by the manufacturer. For + 0.05 mm on the new source diameter, the uncertainty was less than 1.34% for channels 1 and 2, and was 1.42% for channel 3 in the ranges of − 10 cm ≤ z ≤ 10 cm and 0 ≤ y ≤ 10 cm, respectively. For − 0.05 mm on the new source diameter, the uncertainty was less than 1.24% for channels 1 and 2, and was 1.35% for channel 3, respectively. For the ^60^Co spectrum, the dominant interaction is the Compton effect. All the tabulated cross sections data are well established. Thus, as suggested in previous works [24, 29], the uncertainty in cross sections and energy spectrum were negligible. Thus, the quadrature sum of these three terms gives a total uncertainty of 2.19% for channels 1 and 2, and 2.29% for channel 3, respectively (type A and type B).

## Conclusion

In this study, the full dosimetric parameters for the new GZP3 ^60^Co sources were obtained using the Monte Carlo Geant4 and EGSnrc codes, based on the dose-calculation formalism recommended by the AAPM TG43 and TG43U1 reports. The dose-rate constant, radial dose function, anisotropy function and away–along dose-rate distributions were calculated for the new GZP3 ^60^Co sources. Furthermore, the dosimetric parameters of the new GZP3 ^60^Co source were not previously published. These dosimetric datasets can be used as input data and in the quality control of the TPS calculations.

## Data Availability

The data are fully available without restriction in a public repository (Dryad). The reference treatment plans and corresponding perturbed plans were archived in the Informatic System of Sichuan Cancer Hospital.
